# Laboratory data on the interfacial tension, viscosity, and density of two naphthenic acids in n-hexadecane across varying temperatures and concentrations

**DOI:** 10.1016/j.dib.2025.111322

**Published:** 2025-01-25

**Authors:** Mohammad Sarlak, Alan J. McCue, Yukie Tanino

**Affiliations:** a School of Engineering, University of Aberdeen, Aberdeen AB24 3UE, United Kingdom; b Core Specialist Services, Aberdeen, AB22 8AA, United Kingdom; c Advanced Centre for Energy and Sustainability (ACES), School of Natural and Computing Sciences, University of Aberdeen, Aberdeen, AB24 3UE, Scotland, United Kingdom

**Keywords:** Interfacial properties, Thermophysical properties, Pendant drop method, Stearic acid, Cyclohexanepentanoic acid

## Abstract

This paper presents oil-brine interfacial tensions measured using the pendant drop method at temperatures ranging from T = 25.0 to 60.0°C. Test oils were solutions of stearic acid or cyclohexanepentanoic acid in n-hexadecane at concentrations ranging from 0 to 2200 mg/L; the test brine was 5 wt.% NaCl and 1 wt.% KCl in deionized water for all measurements. Also presented are viscosity and density measurements by rotational viscometry at temperatures ranging from T = 20.0 to 80.0°C.

Specifications Table

**Table:** 

Subject	surface chemistry
Specific subject area	interfacial properties, thermophysical properties
Type of data	raw videos, Excel tables (interfacial tension values are not reported in the Excel file if the drop volume is smaller than or equal to 95 % of its initial volume.) Table, Figure etc. Raw, Filtered, etc.
Data collection	interfacial tension: pendant drop method using First Ten Angstroms FTA100, with FLIR camera USB 2.0-FMVU-03MTM-CS (0.3 MP, 60 FPS, Aptina MT9V022, Mono) and FTA Video Drop Shape Analysis software. viscosity and density: rotational viscometry using Anton Paar SVM^TM^ 3000
Data source location	With corresponding author (Yukie Tanino) at University of Aberdeen, Aberdeen, UK
Data accessibility	Data is with this article. Repository name: Pure Data identification number: 10.20392/f3bfa822-9924-4071-85e8-1ec9f88513d5 Direct URL to data: https://doi.org/10.20392/f3bfa822-9924-4071-85e8-1ec9f88513d5 Instructions for accessing these data: n/a
Related research article	

## 1 Value of The Data

These data are of value to the scientific community because of:

• The interfacial tension (σ) data can be used to determine the capillary number of two-phase flows of stearic acid or cyclohexanepentanoic acid in n-hexadecane and the test brine system.
• The data can be used to describe the impact of concentration and temperature on the interfacial tension of naphthenic acids in n-hexadecane and brines.
• The data may be compared against measurements acquired using different techniques, e.g., du Noüy rings.
• Raw data may be used as test images to develop and validate algorithms for extracting interfacial tensions from drop shapes.

## 2 Background

Naphthenic acids are a group of carboxylic acids typically found in crude oil, particularly in heavy crude and bitumen. Naphthenic acids can significantly influence σ between oil and water. This is important for oil recovery and crude oil refining. Solutions of naphthenic acids in alkane hydrocarbons are widely used as analogues of crude oil to render laboratory rock samples mixed wet (e.g., Refs. [[1], [2], [3], [4], [5]]). Tanino and Blunt in 2013 [1] found that organic acids dissolved in oil phase impact on the remaining oil saturation by waterflooding in Indiana limestone. Christensen and Tanino in 2017 [2,3] noted organic acids dissolved in oil phase alter the Indiana limestone wettability by measuring the advancing contact angle. Al-Shirawi et al. in 2021 [4] observed that wettability of strongly water wet carbonate surfaces altered toward oil-wetness due to aging in the oil model (stearic acid was added to oil phase). Subsequent displacement of these oils by an aqueous phase injected into the rock is largely controlled by the capillary number and mobility ratio, which are functions of σ between the two liquids and their viscosities.

## 3 Data Description

The dataset comprises (a) oil-brine σ measured using the pendant drop method and (b) thermophysical properties of the test fluids at varying temperatures. In total, 45 pendant drops of varying concentrations of stearic acid or cyclohexanepentanoic acid in n-hexadecane with brine at three temperatures were analysed; raw images (at least five per drop, taken a minimum of 0.20 seconds apart) can be found in the Supplementary Materials. The IFT values extracted from the images are reported in the Excel file “IFT.xlsx” as a function of concentrations and temperatures. The images for each fluid set are also provided on the repository. Density and dynamic viscosity of all test fluids measured at temperatures ranging from 20.0 to 80.0°C are listed in the Excel file “thermophysical.xlsx”. The description of the data available in the repository is presented in Table 1. Table 2 also presents empirical models that describe the temperature dependence of the density and viscosity*.*

**Table 1:** Data description for all data set available in the repository.

solute	concentration [mg/L]	Temperature [°C]	Data Type	Variable	Data files
**–**	**–**	25, 40, 60	Images	IFT	1-_Pure_Hexadecane.zip
stearic acid	300	25, 40, 60	Images	IFT	2-_1st_SA_300_.zip
stearic acid	610	25, 40	Images	IFT	3-_2nd_SA_610_.zip
stearic acid	888	30, 40, 60	Images	IFT	4-_3rd_SA_888_.zip
stearic acid	1238	30, 40, 60	Images	IFT	5-4th_SA_1238_.zip
stearic acid	1516	30, 40, 60	Images	IFT	6-_5th_SA_1516_.zip
stearic acid	1912	30, 40, 60	Images	IFT	7-_6th_SA_1832_.zip
stearic acid	2184	30, 40, 60	Images	IFT	8-_7th_SA_2158_.zip
CHPA	400	30, 40, 60	Images	IFT	9-1st_CHPA_400_.zip
CHPA	650	30, 40, 60	Images	IFT	10-2nd_CHPA_650_.zip
CHPA	886	30, 40, 60	Images	IFT	11-3rd_CHPA_886_.zip
CHPA	1232	30, 40, 60	Images	IFT	12-4th_CHPA_1232_.zip
CHPA	1578	30, 40, 60	Images	IFT	13-5th_CHPA_1578_.zip
CHPA	1912	30, 40, 60	Images	IFT	14-6th_CHPA_1912_.zip
CHPA	2158	30, 40, 60	Images	IFT	15-7th_CHPA_2158_.zip
stearic acid, CHPA	0 to ≈2200	25, 30, 40, 60	Excel	IFT	IFT.xlsx
stearic acid, CHPA	0 to ≈2200	20, 25, 30, 35, 40, 50, 60, 70, 80	Excel	Density, dynamic viscosity	thermophysical.xlsx

**Table 2:** Test oils and lines of best fit in the least squares sense to density and viscosity measurements (Excel file thermophysical.xlsx). The best-fit functions were previously used to evaluate the density and viscosity of n-hexadecane and varying concentrations of stearic acid or CHPA in n-hexadecane [7]. The solvent is n-hexadecane. T is temperature in units of°C; R^2^ is the coefficient of determination.

solute	concentration [mg/L]	density (kg/m^3^)	viscosity (µPa.s)
**–**	**–**	−0.6942T + 787.92, R^2^=1.0000	−1.715ln(T) + 8.5351, R^2^=0.9909
stearic acid	300	−0.6943T + 787.99, R^2^=1.0000	−1.716ln(T) + 8.5378, R^2^=0.9913
stearic acid	610	−0.6945T + 788.04, R^2^=1.0000	−1.681ln(T) + 8.4325, R^2^=0.9940
stearic acid	888	−0.6948T + 788.11, R^2^=1.0000	−1.681ln(T) + 8.4354, R^2^=0.9940
stearic acid	1238	−0.6954T + 788.21, R^2^=1.0000	−1.681ln(T) + 8.4389, R^2^=0.9940
stearic acid	1516	−0.6951T + 788.23, R^2^=1.0000	−1.681ln(T) + 8.4418, R^2^=0.9940
stearic acid	1912	−0.6951T + 788.30, R^2^=1.0000	−1.682ln(T) + 8.4457, R^2^=0.9940
stearic acid	2184	−0.6960T + 788.38, R^2^=1.0000	−1.682ln(T) + 8.4488, R^2^=0.9940
CHPA	400	−0.6940T + 788.00, R^2^=1.0000	−1.679ln(T) + 8.4242, R^2^=0.9940
CHPA	650	−0.6941T + 788.05, R^2^=1.0000	−1.680ln(T) + 8.4269, R^2^=0.9940
CHPA	886	−0.6931T + 788.06, R^2^=1.0000	−1.680ln(T) + 8.4292, R^2^=0.9939
CHPA	1232	−0.6928T + 788.12, R^2^=1.0000	−1.680ln(T) + 8.4319, R^2^=0.9938
CHPA	1578	−0.6925T + 788.17, R^2^=1.0000	−1.680ln(T) + 8.4342, R^2^=0.9938
CHPA	1912	−0.6919T + 788.23, R^2^=1.0000	−1.681ln(T) + 8.4373, R^2^=0.9937
CHPA	2158	−0.6923T + 788.32, R^2^=1.0000	−1.681ln(T) + 8.4397, R^2^=0.9937

## 4 Experimental Design, Materials and Methods

### 4.1 Test fluids

The aqueous phase was 5 wt.% NaCl and 1 wt.% KCl [6] in deionized water in all experiments. Prior to use, the aqueous phase was filtered through one sheet of filter paper (0.25 µm particle retention, Whatman) to remove any undissolved impurities, then degassed under vacuum (Edwards RV8).

Test oils were prepared by adding the desired mass of stearic acid or cyclohexanepentanoic acid (CHPA) to a 100 mL volumetric flask with a lid, then filling the flask with n-hexadecane. Fifteen oils were considered: n-hexadecane (Fisher Scientific, 99 %), stearic acid (Sigma-Aldrich, ≥98.5 %) in n-hexadecane (Fisher Scientific, 99 %) at seven concentrations between 300 and 2184 mg/L, and CHPA (Sigma-Aldrich, 98 %) in n-hexadecane (Fisher Scientific, 99 %) at seven concentrations between 400 and 2158 mg/L. The aqueous phase and test oils were mixed and agitated at 1:1 volume ratio, then stored in stationary glass for 72 hours prior to σ measurements to ensure equilibrium.

### 4.2 Density and dynamic viscosity measurements

Dynamic viscosities and densities of each test fluid listed in Table 2 were measured by rotational viscometry at temperatures between T = 20.0 and 80.0°C (Anton Paar SVM^TM^ 3000). One sample was loaded into the instrument and the density and viscosity were measured at pre-selected temperatures. A fresh volume of the same test fluid was injected, and the measurements repeated. Following this procedure, measurements of viscosity and density were acquired on at least two samples at each temperature. The instrument uncertainty in density and viscosity measurements are ±1 kg/m^3^ and ±0.001 µPa.s respectively.

### 4.3 σ measurements

σ between the aqueous phase and each test oil listed in Table 2 was measured using the pendant drop method (FTA100 from First Ten Angstrom). At least five independent runs were performed for each oil and brine set, where each run comprised:

1. Upward injection of the test oil into the chamber until to achieve a suitable height of the drop. The suitable height of the drop is crucial in the pendant drop method for measuring σ due to shape stability, measurement precision, gravity effect, evaporation and instability. A small drop may not have a stable shape, leading to inaccuracies in measurements. If the drop is not within the optimal height range, it may lead to a less accurate curvature measurement, impacting the σ calculation. The influence of gravity becomes more pronounced at greater heights, which can alter the drop's shape and result in deviations from the ideal spherical shape used in σ calculations. If the drop is too high, evaporation may occur more rapidly, which can also affect the volume of the drop, leading to inconsistent results.
2. The circulating water bath was set to varying temperatures from T = 20.0 and 80.0°C.
3. A sequence of still images were acquired.

### 4.4 Apparatus preparation (cleaning)

The sample chamber and flow lines were rinsed with the test brine. The chamber was filled with the test brine. The syringe was filled with the test oil. The camera was adjusted until it was in focus. A dispensed needle was passed through the base of the chamber to allow the buoyant oil to form a pendant drop.

Since σ is very sensitive to contamination, the needle, the syringe and chamber were cleaned with dichloromethane (DCM; Fisher Scientific, ≥99.8 %) and methanol (Fisher Scientific, ≥99.8 %) and dried in air prior to use.

### 4.5 Dispensing the drop and image acquisition

σ was measured at T = 25.0 or 30.0, 40.0, and 60.0°C using a circulating water bath.

1. The circulating water bath was programmed to the desired temperature and the temperature of the chamber monitored as a function of time. Approximately 10 minutes was required for the temperature in the chamber to reach the temperature set for the circulating water bath. A circulating water bath provides a uniform temperature environment, minimizing fluctuations. Temperature fluctuations affect the viscosity and density of the liquids involved. This stability is essential for consistent σ readings. Circulating water ensures even heat distribution, preventing localized hot or cold spots that could alter the behavior of the drop or the properties of the fluids. Circulating water allows sufficient time for the system to reach thermal equilibrium ensures that all components are at the same temperature, which is crucial for precise σ measurements. Using a circulating water batch provides consistent temperature control across multiple experiments, increasing the reproducibility of results, making it easier to compare different measurements and draw reliable conclusions.
2. Once constant temperature was achieved in the chamber, the test oil which is at room temperature was manually dispensed at a rate of 2 mL/min into the brine-filled chamber upward through a U shape needle (ID 0.718 mm) using a standard pipette syringe. The injection was continued until the height of the drop was sufficiently large for the pressure difference between the top and bottom to distort the drop (σ cannot be determined for a spherical drop); the largest drop volumes were 31.22, 31.10, and 22.10 µL for test oils without acid, with stearic acid, and with CHPA, respectively.
3. Once the drop was distorted by gravity, its profile was captured in a sequence of still images over 5 to 30 minutes by a high-resolution camera.

At least five replicate runs were performed for each combination of solute concentration and temperature. The FTA100 uncertainty in measuring σ is ±0.01 mN/m. For each run, the apparatus was cleaned as per above, the chamber was filled with fresh test brine, the desired temperature was established in the chamber, and an oil drop was dispensed.

### 4.6 Determination of σ using the FTA software

**Fig. 1:**
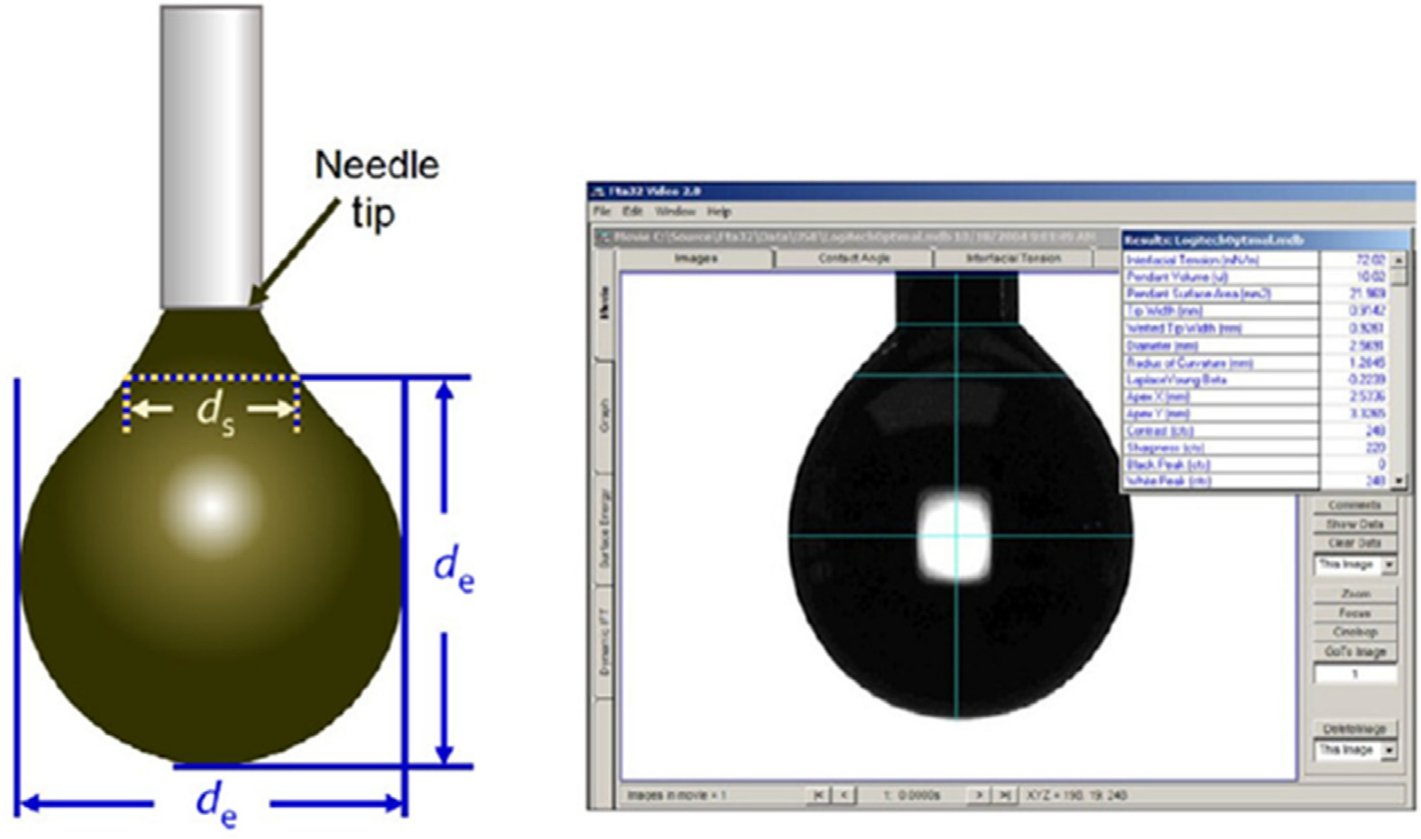
Pendant drop method (First Ten Angstroms).

The shape of a stationary pendant drop is described by the Young Laplace equation,

$$\sigma \left[\frac{1}{R_{1}\left(z\right)}+\frac{1}{R_{2}\left(z\right)}\right]=\Delta P\left(z\right)=\Delta P\left(z=0\right)-\left(\rho_{d}-\rho_{\text{out}}\right)\text{gz}$$

where $\Delta P\left(z\right)=P_{d}\left(z\right)-P_{\text{out}}\left(z\right)$, $P_{d}$ is the pressure inside the drop, $P_{\text{out}}\left(<P_{d}\right)$ is the pressure outside the drop, g is the acceleration due to gravity, and z is the vertical coordinate with z = 0 defined at the apex of the drop. The above equation can be written as a set of differential equations in terms of the arc length along the interface, which in turn can be solved numerically (see, e.g., Berry et al. 2015 [8]). The software extracted one value of σ from each image as

$$\sigma =\frac{g\;\left(P_{\text{out}}-P_{d}\right){\;d}_{e}^{2}}{H}$$

where d_e_ is the maximum drop diameter, d_s_ is the diameter of the cross-section of the drop at z = - d_e_, and H is a shape factor that is a function of d_e_/d_s_ (Fig. 1).

## Limitations

None.

## Ethics Statement

The authors have read and follow the ethical requirements for publication in Data in Brief and confirming that the current work does not involve human subjects, animal experiments, or any data collected from social media platforms.

## CRediT Author Statement

**Mohammad Sarlak:** Conceptualization, Investigation, Methodology, Validation, Writing – original draft, Writing – review and editing. **Alan J. McCue:** Writing – review and editing. **Yukie Tanino:** Conceptualization, Methodology, Resources, Supervision, Funding acquisition, Writing–review and editing.

## Data Availability

PUrEData from laboratory measurements of interfacial tension, viscosity, and density of two naphthenic acids in n-hexadecane at varying temperature and concentrations (Original data).
